# Mismatch Negativity in Rat Auditory Cortex Represents the Empirical Salience of Sounds

**DOI:** 10.3389/fnins.2018.00924

**Published:** 2018-12-17

**Authors:** Tomoyo Isoguchi Shiramatsu, Hirokazu Takahashi

**Affiliations:** Research Center for Advanced Science and Technology, The University of Tokyo, Tokyo, Japan

**Keywords:** mismatch negativity, microelectrode array, classical conditioning, empirical salience, schizophrenia

## Abstract

Mismatch negativity (MMN) is an N-methyl-D-aspartic acid-mediated component and thus has been widely considered a major candidate biomarker of schizophrenia. However, at present, no direct evidence has linked the MMN response and aberrant salience processing reported in schizophrenia patients, i.e., whether MMN represents empirical salience of the deviant stimulus. To test the eligibility of the MMN response as a biomarker of schizophrenia, we hypothesized and verified that the MMN in the rat auditory cortex encodes empirical salience, which depends on both empirical rarity of sound and association of sound with salient events through classical conditioning. We exposed rats to sound or appetitive or aversive classical conditioning and extinction training of aversive conditioning, then recorded MMN from the auditory cortex. We focused on the frequency-dependent asymmetry of the MMN amplitude; increment frequency changes elicit asymmetrically larger MMN amplitudes than do decremental frequency changes. We found that sound exposure reversed this asymmetry in rats, indicating that MMN encodes the empirical rarity of deviant sounds. The MMN amplitude was larger in the appetitive or aversive classical conditioning groups, and smaller after extinction training following aversive conditioning. These results indicate that the MMN response reflects the empirical salience of sound. The present work first linked MMN with empirical salience processing and expands the possibilities of MMN as a strong clinical biomarker of schizophrenia.

## Introduction

Since its first description in 1978, the mismatch negativity (MMN) response has been widely investigated in the context of the memory-based deviance detection system. MMN is a component of the auditory evoked potential (AEP) and occurs in response to a deviant stimulus embedded within repetitive standard stimuli (Näätänen et al., 1978). As one of the most interesting characteristics of MMN, accumulating evidence has indicated that MMN cannot be fully explained by the mere effect of neural adaptation, i.e., stimulus-specific adaptation (SSA) to the repeated standard stimuli. Although SSA and MMN are often observed in the same paradigm, i.e., the oddball paradigm (Shiramatsu et al., 2013; Escera and Malmierca, 2014), some studies have highlighted their differences; categorical change such as musical chord consonance and grammatical error can elicit MMN but not SSA (Alho et al., 1996; Pulvermüller and Shtyrov, 2003; Näätänen et al., 2007), and a recently-suggested control paradigm distinguished MMN from SSA (Farley et al., 2010; Fishman and Steinschneider, 2012; Shiramatsu et al., 2013). In addition, successful discrimination learning of unfamiliar sounds, e.g., non-native vowels or pattern of Mores signal, enhanced MMN, supporting the view that MMN is not fully mediated by adaptation but reflects an experience-dependent deviance detection system (Menning et al., 2002; Kujala et al., 2003; Näätänen et al., 2007). However, despite numerous studies using discrimination learning, few have applied classical conditioning of a particular sound and investigated what kind of sound information is represented in MMN.

To address this question, we focused on the clinical significance of MMN for the study of schizophrenia and the recent framework of this disease, i.e., the “aberrant salience hypothesis.” A reduced MMN amplitude in patients with schizophrenia has been repeatedly reported (Shelley et al., 1991; Baldeweg et al., 2004) and the blockage of N-methyl-D-aspartic acid (NMDA) receptors mimicked this reduction of MMN, which correlates with the severity of some symptoms of schizophrenia (Kreitschmann-Andermahr et al., 2001; Umbricht et al., 2002; Tikhonravov et al., 2010; Shiramatsu et al., 2013). Based on these findings, MMN has been widely considered a major candidate biomarker for the disease. Besides, the “aberrant salience hypothesis of schizophrenia” framework has recently emerged, based on the cognitive and behavioral impairments of the patients such as experience-based or empirical predictions (Hemsley, 1998, 2005a,b; Kapur, 2003; Keefe et al., 2011; Nelson et al., 2014; Uddin, 2015). A putative neural substrate of this aberrant empirical salience is the saliency network including the insula (Ellison-Wright et al., 2008; Glahn et al., 2008), which is also activated in response to deviants in the oddball paradigm (Downar et al., 2000, 2002; Sridharan et al., 2008; Palaniyappan et al., 2013). Altogether, these findings suggest that MMN represents the empirical saliency of deviant stimuli, and thus can be a reliable biomarker for the aberrant salience hypothesis of schizophrenia (Nelson et al., 2014).

Here, we tested the eligibility of MMN as a biomarker of schizophrenia, by focusing on the relationship between MMN and empirical salience. We hypothesized that the MMN response in the rat auditory cortex encodes the empirical salience of sound, which depends on both the (i) empirical rarity of sound and (ii) association of sound with salient events through classical conditioning. For the first hypothesis, we focused on “frequency-dependent asymmetry” of the MMN amplitude; even when the contrast between standard and deviant stimuli is fixed, incremental frequency changes elicit larger MMN amplitudes than do decremental frequency changes (Peter et al., 2010; Isoguchi et al., 2011), but the behavioral meaning of this effect has not been elucidated. Based on natural environmental noise, where low-frequency components are dominant, we hypothesized that this asymmetry property of the MMN amplitude may reflect the empirical rarity of sound acquired from surrounding noises. Therefore, we first investigated whether exposure to a specific sound, which decreases its empirical rarity, inverts the asymmetry of the MMN amplitude. Second, we manipulated the empirical salience of a sound by associating or dissociating it with a specific event using appetitive or aversive classical conditioning and extinction of the aversive conditioning and investigated how these manipulations change the MMN amplitude.

## Materials and Methods

This study was conducted in strict accordance with the “Guiding Principles for the Care and Use of Animals in the Field of Physiological Science” published by the Japanese Physiological Society. The experimental protocol was approved by the Committee on the Ethics of Animal Experiments at the Research Center for Advanced Science and Technology, the University of Tokyo (Permit Number: RAC130107). All surgery was performed under isoflurane anesthesia, and all efforts were made to minimize the suffering of animals. After the experiments, animals were euthanized with an overdose of pentobarbital sodium (160 mg/kg, i.p.). The raw data supporting the conclusions of this manuscript will be made available by the authors, without undue reservation, to any qualified researcher.

### Subjects

Thirty-five male Wistar rats at postnatal weeks 8–10 and with a body weight of 230–300 g were used in this study. Five groups were tested (each group *n* = 7), as follows: (i) naive group, where neither exposure nor conditioning was conducted; (ii) exposed group; (iii) appetitive conditioning group; (iv) aversive conditioning group; and (v) extinction group.

### Auditory Exposure, Classical Conditioning, and Extinction

**Figure 1:**
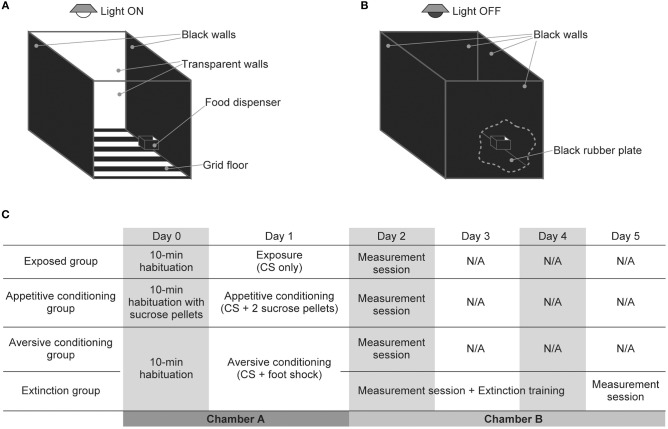
Experimental setup and design of auditory exposure, classical conditioning, and extinction. **(A,B)** Schema of the experimental setup for **(A)** auditory exposure and classical conditioning and **(B)** auditory extinction and the measurement session. There was a food dispenser in one of the black walls to supply sucrose tablets for rats in the appetitive conditioning group. Three elements changed in the measurement session and auditory extinction. (i) The light above the experimental chamber was turned off, (ii) the grid floor that delivered the foot shock was covered with a black rubber plate, and (iii) two transparent walls of the chamber were replaced with black walls. These changes prevented contextual conditioning and also emphasized the contrast between the white rats and black walls and floor, which enabled easier behavioral measurement from camera-captured images. **(C)** Schedule for the exposure, classical conditioning, and extinction.

Behavioral experiments were performed in a custom-made experimental chamber (OPEZ-3001; O'hara & Co. Ltd., Tokyo, Japan) measuring 35 × 35 × 35 cm (Figures 1A,B). The chamber had two black and two transparent acrylic walls and a black-metallic grid floor. It was equipped with a food dispenser on one of the black walls and with a speaker (Technics EAS-10TH800; Matsushita Electric Industrial Co. Ltd., Osaka, Japan for the exposed, appetitive conditioning, and aversive conditioning groups; DDL-RT16C; Alpine Electronics, Inc., Tokyo, Japan for the extinction group) on the ceiling. Prior to the experiments, acoustic calibration was performed for a 16 kHz pure tone with a 1/4-inch microphone (Type 4939; Brüel & Kjaer, Nærum, Denmark) and spectrum analyzer (CF-5210; Ono Sokki Co., Ltd., Kanagawa, Japan) at the center of the arena at a height of 10 cm in the frequency range of 125 Hz−64 kHz.

Before initiating behavioral procedures, the rats in the appetitive conditioning group were placed on a food-deprivation schedule to reduce weight to 85% of their free-feeding baseline weight. One day prior to the exposure or conditioning (Day 0), rats in the exposure, appetitive conditioning, aversive conditioning, and extinction groups were placed in the experimental chamber for 10 min. During this habituation, rats in the appetitive conditioning group were also served sucrose pellets from the food dispenser (Figure 1C). On the next day (Day 1), exposure or classical conditioning was conducted. The conditioned stimulus (CS) was a pure tone with a frequency of 16 kHz, an intensity of 60 dB SPL (sound pressure level in decibels with respect to 20 μPa), and a duration of 10 s. The last 1 s of the CS was associated with an unconditioned stimulus (US), which consisted of two sucrose pellets for the appetitive conditioning group or an electrical foot shock (0.3 mA, 1 s) for the aversive conditioning and extinction groups. For the exposed group, no US was associated with the CS. These CS or CS-US pairs were presented 40 times (or 20 times for one aversive-conditioned rat) with a pseudo-random inter-stimulus interval ranging from 1 to 4 min. The total time of the exposure and conditioning was approximately 120 min.

The day after conditioning (Day 2), a measurement session was conducted whereby the approaching times to the dispenser and the freezing times were measured to confirm appetitive and aversive conditioning, respectively. In order to differentiate the contexts of conditioning from those of the measurement session and to prevent contextual conditioning, the chamber used for the behavioral measurements had four black walls and a black hard rubber plate on the floor, and the light of the chamber was turned off [(Funamizu et al., 2013); Figure 1B]. To evaluate appetitive conditioning, an infrared sensor in the food dispenser measured the approaching time (the time from CS onset to first nose poking of the dispenser) of rats in the exposed and appetitive conditioning groups. We started to deliver the 60 s CS when the rats were in the opposite half of the arena to the dispenser, and the approaching time measurements were censored at 60 s with or without poking. To evaluate aversive conditioning and extinction, the freezing time of rats in the aversive-conditioning and extinction groups were measured for 60 s under silence or with presentation of the CS (Funamizu et al., 2013). A camera placed above the chamber captured images every 0.5 s, and images were binarized to identify the position of the rats and background as white and black areas, respectively. Freezing time was then defined as an accumulated period, during which image-by-image differences did not reach an empirical threshold. The threshold was determined such that image-based and human-observation-based freezing times showed a concordance of 90% or more (Moita et al., 2003).

For 3 days following the aversive conditioning (Days 2–4), extinction training was also applied to the rats in the extinction group (Figure 1C). In one extinction session, the CS was presented five times with a pseudo-random inter-stimulus interval ranging from 3 to 5 min. For daily extinction training, four behavioral measures of the freezing time and three extinction sessions were alternately conducted; the total time was approximately 120 min. On the following day (Day 5), the final freezing time measurement was conducted.

### Neural Recordings

Rats were anesthetized with isoflurane in conjunction with air (3% at induction and 1–2% for maintenance) and were held in place with a custom-made head-holding device. Atropine sulfate (0.1 mg/kg) was administered subcutaneously at the beginning and at the end of surgery to reduce the viscosity of bronchial secretions. A heating blanket was used to maintain body temperature at approximately 37°C. The skin was incised at the beginning of the surgery under local anesthesia with xylocaine (0. 3–0.5 ml). A needle electrode was subcutaneously inserted into the right forepaw and used as a ground. A small craniotomy was made near the bregma landmark to embed a 0.5 mm thick integrated circuit socket as a reference electrode, with an electrical contact to the dura mater. The right temporal muscle, cranium, and dura overlying the auditory cortex were surgically removed, and the exposed cortical surface was perfused with saline to prevent desiccation. Cisternal cerebrospinal fluid drainage was performed to minimize cerebral edema. The right eardrum, i.e., ipsilateral to the exposed cortex, was ruptured and waxed to ensure unilateral sound inputs from the ear contralateral to the exposed cortex. Respiratory rate, heart rate, and hind-paw withdrawal reflexes were monitored throughout the experiment to maintain an adequate and stable anesthetic level.

A surface microelectrode array was used to record AEPs from the auditory cortex (Shiramatsu et al., 2013). The microelectrode array was constructed on a flexible polyimide substrate to conform to the curvature of cortical surface, with a grid of 10 × 7 recording sites within an area of 4.5 × 3.0 mm. Each recording site was 50 × 50 μm, and the electrode impedance was approximately 400 kΩ under 1 kHz, 0.1 V sinusoidal waves. Neural signals were obtained with an amplification gain of 1,000, a digital filter bandpass of 0.3–500 Hz, and a sampling frequency of 1 kHz (Cerebus Data Acquisition System; Cyberkinetics Inc., Salt Lake City, USA). A speaker (Technics EAS-10TH800; Matsushita Electric Industrial Co. Ltd., Osaka, Japan) was positioned 10 cm from the left ear, i.e., contralateral to the exposed cortex. Test stimuli were calibrated at the pinna with the microphone and spectrum analyzer, which is also used in the calibration for the behavioral experiments.

First, we mounted the surface microelectrode array on the temporal cortex and mapped the click-evoked responses to identify the location of the auditory cortex. Clicks were presented 60 times to obtain a grand average of click-evoked responses. The microelectrode array covered the entire auditory cortex, including the core and belt regions (Takahashi et al., 2005; Shiramatsu et al., 2013). Our previous data showed that the tone-elicited MMN spreads equally over the core and belt regions (Shiramatsu et al., 2013); thus, we first determined the putative auditory region, i.e., the core and belt regions (Figure 1). The primary and anterior auditory fields (A1 and AAF) were responsive to clicks and thus were included in the core region, while other tone responsive areas, including the ventral and suprarhinal auditory fields (VAF and SRAF), were considered as the belt region.

Tone-elicited MMN responses in the putative auditory region were then obtained using an auditory oddball paradigm. Test stimuli were tone bursts with a 60 dB SPL plateau and a 100 ms duration with 5 ms rise/fall times. The inter-stimulus interval was 600 ms. In the oddball paradigm, two tones with differing frequencies served as either a frequent standard (*p* = 0.9, i.e., 540 times) or a rare deviant (*p* = 0.1, i.e., 60 times). The tone frequencies of standard and deviant tones were swapped for the two oddball sessions. In this study, one tone frequency was fixed at 16 kHz, i.e., the CS in the exposure and classical conditioning groups, and the other was selected according to the two following stimulus parameters: (i) relative height to 16 kHz, i.e., low or high, to investigate the frequency-dependent asymmetry of the MMN amplitude, and (ii) frequency separation (Δf), i.e., 1/3, 2/3, and 1 octave, to cover the audibility range of rats; thus, six tone pairs were tested in total (Table 1).

**Table 1:** Sound frequency of tone pairs used in the experiments.

	**Relative height to 16 kHz**	**Frequency separation (octave)**	**Tone A (kHz)**	**Tone B (kHz)**
Pair 1		1	8.0	16
Pair 2	Lower frequency	2/3	10	
Pair 3		1/3	13	
Pair 4		1/3	20	
Pair 5	Higher frequency	2/3	25	
Pair 6		1	32	

### Identification of Auditory Area

**Figure 2:**
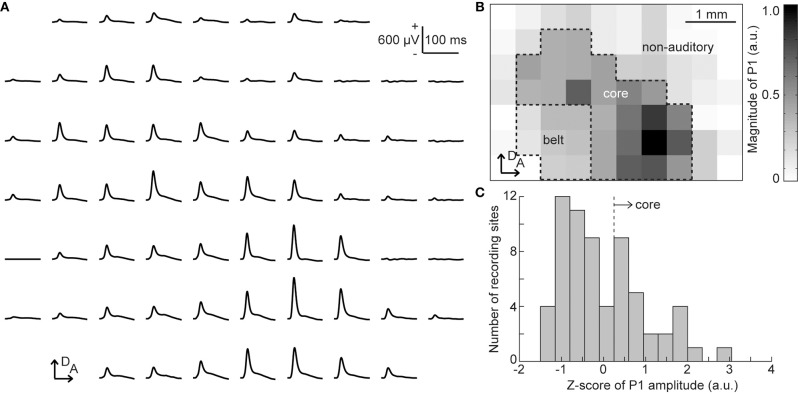
Identification of the putative auditory area. **(A)** Representative mapping of grand-averaged auditory evoked potential (AEP) waveforms for 60 clicks. Each AEP waveform is approximately aligned in the spatial coordinates of the 64 sites of the surface microelectrode array. At each recording site, the P1 amplitude was quantified as the maximum potential within 50 ms from the stimulus onset. **(B)** Spatial distribution of the click-evoked P1. The gray level corresponds to the P1 amplitude measured at each electrode in the array. The putative core region and belt region were determined according to P1 amplitudes, and other sites comprised the non-auditory region. Both the core and belt regions were considered as the auditory area in this study. **(C)** Histogram of the z-scores of P1 amplitude. The core region was defined as the recording sites where z-scores had larger values than the automatically-calculated threshold (broken line). A, anterior; D, dorsal.

From the click-elicited AEPs, we determined the putative auditory regions of each animal, i.e., the core and belt regions, according to the following procedure (Figure 2). First, we quantified the amplitude of the click-elicited P1 as the maximum potential within 50 ms from click onset. The histogram of the z-scores of the P1 amplitude at all recording sites was then calculated to determine the responsive sites, i.e., the core region exhibiting large click-elicited P1 amplitudes. The threshold of z-scores was defined as the maximal value of second-order differentials of a z-scores histogram with a maximum value, i.e., the inflection point (Guo et al., 2012). The core region was defined as the recording sites at which z-scores had larger values than the threshold, and the recording sites enclosed in the ventral and posterior border of the core region were categorized as the belt region. Our previous report showed that pure-tone elicited P1 and MMN responses are both generated in the auditory cortex (Shiramatsu et al., 2013); thus, we grouped the core and belt regions together as the putative auditory area.

### Neural Characteristics

**Figure 3:**
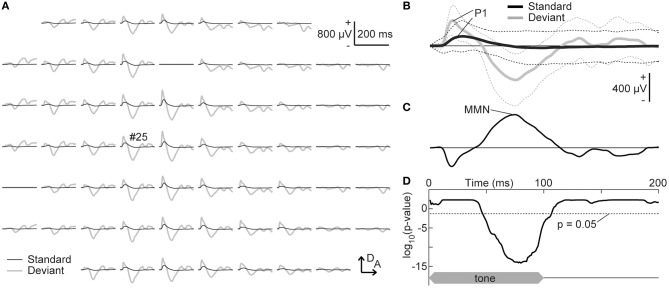
Tone-evoked mismatch negativity (MMN). **(A)** Representative mapping of grand-averaged standard (black) and deviant (gray) auditory-evoked potentials (AEPs). The frequency of these stimuli was 16 kHz, which was paired with 8.0 kHz in the oddball paradigm. **(B)** AEPs from an indicated recording site (#25). The mean (solid lines) ± standard deviation (broken lines) of the 540 (standard) and 60 (deviant) recordings are given. The maximum amplitude of the P1 and MMN in the auditory area was used for the analysis. **(C)** Difference waves obtained by subtracting the deviant AEPs from the standard AEPs. **(D)** Significance level under the null hypothesis that deviant AEPs are larger than standard AEPs at a given post-stimulus latency time (one-sided Wilcoxon rank sum test with Bonferroni correction for 200 comparisons). The time course of stimulus presentation is indicated at the bottom of the inset. A, anterior; D, dorsal.

The grand-averaged deviant AEP was subtracted from the standard AEP response to the same tone frequency, i.e., in the other, second session, and the MMN amplitude was quantified as the maximum potential of this difference wave between 50 and 150 ms from sound onset (Figure 3). Our first hypothesis was that the frequency-dependent asymmetry of the MMN amplitude would reverse by decreasing the empirical rarity of a sound. To evaluate this hypothesis, the asymmetry index of a specific potential was defined as follows:

(1) $$\begin{array}{l} Asymmetry\;index\;\left(AMP\right)=\;\frac{\left(AMP_{high}-AMP_{low}\right)}{\left(AMP_{high}+AMP_{low}\right)} \end{array}$$

where AMP is the amplitude of either the standard P1, deviant P1, or MMN; AMP_high_ and AMP_low_ is the amplitude in response to the higher and lower frequency of each tone pair. The asymmetry index is positive if the higher tone elicits a larger amplitude and negative if the lower tone elicits a larger amplitude.

### Experimental Design and Statistical Analysis

In this study, we examined whether MMN encodes empirical salience, which depends on both the (i) empirical rarity of sound and (ii) association of the sound with salient events through classical conditioning. For the first hypothesis, we focused on the frequency-dependent asymmetry of the MMN amplitude in the naive and exposed groups. Comparisons of MMN and P1 amplitude between incremental change and decremental change and between the naive and exposed groups were assessed by ANOVAs with three factors: relative height of frequency paired with 16 kHz (lower, higher), direction of change (decremental, incremental), and group of rats (naive, exposed). Comparisons of the asymmetry index between incremental change and decremental change and between the naive and exposed groups were also assessed by ANOVAs with two factors: relative height of frequency paired with 16 kHz (lower, higher) and group of rats (naive, exposed). As *post-hoc* comparisons, we used the two-sided Wilcoxon signed-rank test with Bonferroni correction for within-groups comparisons and two-sided Wilcoxon rank-sum test with Bonferroni correction for between-groups comparisons. In addition, the asymmetry index for each pair of frequencies was compared with zero using a two-sided Wilcoxon signed-rank test with Bonferroni correction.

For the second hypothesis, we verified appetitive conditioning by comparing the approaching time between the exposed and appetitive conditioning groups using a log-rank test for the Kaplan-Meier survival function and a two-sided Wilcoxon rank sum test. Aversive conditioning in the aversive conditioning and extinction groups was also verified by comparing the freezing time during the CS presentation and that during silence, using a two-sided Wilcoxon signed-rank test. Then, the MMN amplitudes in the exposed, aversive conditioning, and appetitive conditioning groups were compared using the Games-Howell test for multiple comparisons. Finally, to verify the effect of extinction on MMN, we compared the MMN amplitude between the aversive conditioning and extinction groups using a two-sided Wilcoxon rank sum test. All statistical analyses were performed using MATLAB (Mathworks, Natick, MA, United States).

## Results

### Exposure-Induced Plasticity in MMN

**Figure 4:**
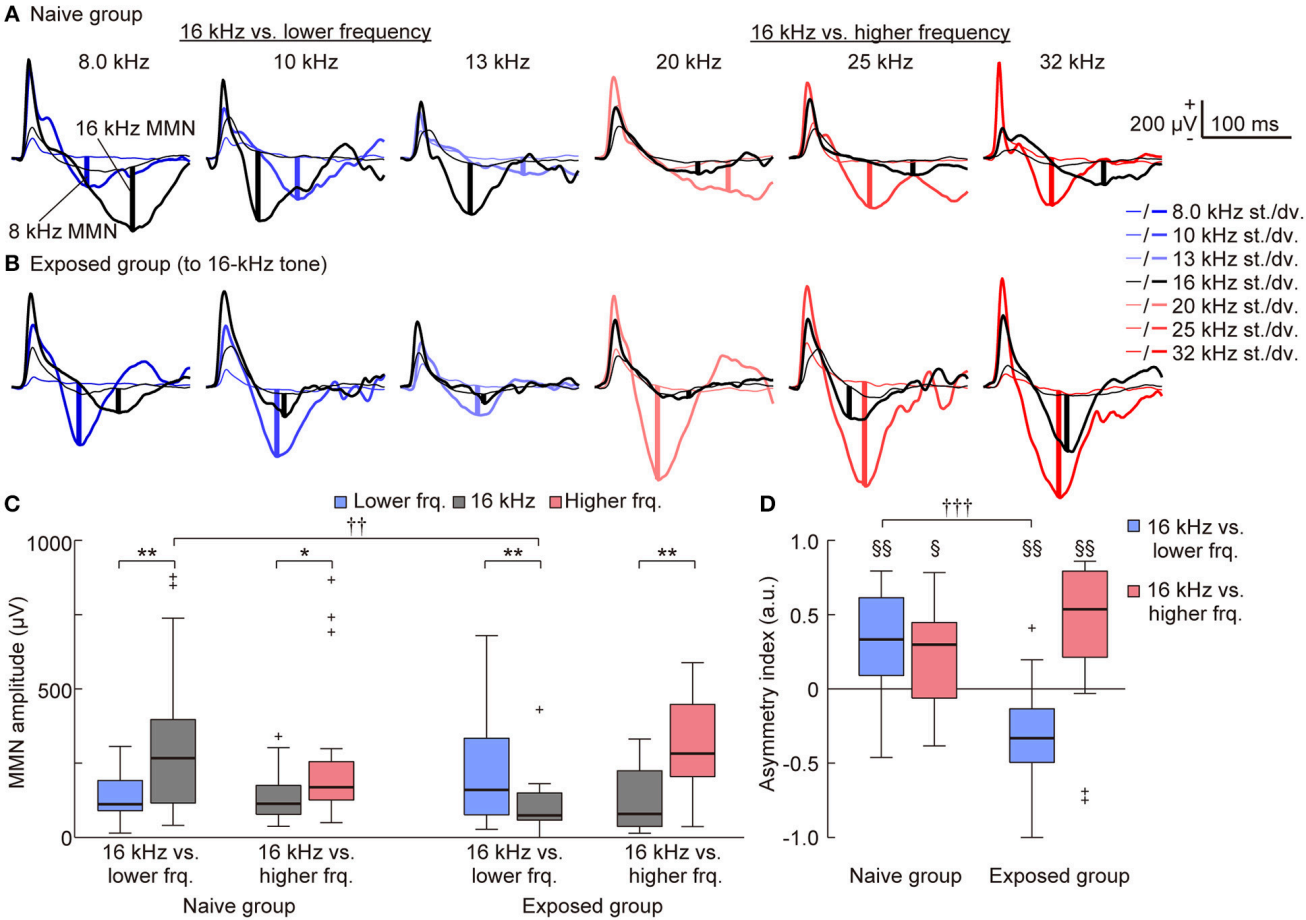
Mere exposure decreased the mismatch negativity (MMN) response. **(A–C)** Representative traces of standard (thin lines) and deviant (bold lines) AEPs of each pair of frequency in **(A)** the naive and **(B)** the 16-kHz exposed group. Black lines indicate AEPs responding to 16 kHz tone, blue and red lines indicate AEPs responding to the lower and higher tones paired with 16 kHz, respectively. The vertical bold lines indicate the MMN amplitude in each trace. **(C)** When pooled in each condition, MMNs to incremental frequency changes were significantly larger than those to decremental changes in the naive group (*n* = 7 per group × 3 tone pairs). After exposure, the MMN responses to incremental frequency changes were significantly smaller than were those to decremental changes when the paired frequency was lower than 16 kHz (16 kHz vs. lower frequency) but remained larger when the paired frequency was higher than 16 kHz (16 kHz vs. higher frequency). **(D)** The asymmetry index of MMN was therefore positive in both conditions in the naive group; however, it became negative in the exposed group when the paired frequency was lower than 16 kHz (*n* = 7 per group × 3 tone pairs). Statistical significance: ^*^p < 0.05; ^**^p < 0.01 (Wilcoxon two-sided signed-rank test), ^††^*p* < 0.01; ^†††^*p* < 0.001 (Wilcoxon two-sided rank-sum test), ^§^*p* < 0.05; ^§§^*p* < 0.01 (Wilcoxon two-sided signed-rank test vs. zero).

With a surface microelectrode array, we epipially mapped AEPs in the auditory cortex of rats and investigated how experience induced plasticity in tone-evoked MMN (Figure 3). To assess whether the frequency-dependent asymmetry of the MMN amplitude would reverse by decreasing the empirical rarity of sound through mere exposure (hypothesis 1), we first compared the frequency-dependent asymmetry of the MMN amplitude between the naive and exposed groups. The representative traces shown in Figures 4A,B provide a quick image of the naive and reversed asymmetry of the MMN amplitude. In the naive group, the higher tone of each tone pair exhibited larger MMN responses than did the lower tone; in the left three traces, where the 16 kHz tone was paired with the lower frequencies, the black bold lines indicate larger MMN for the 16 kHz tone than the MMN for lower tones indicated by blue bold lines, while in the right three traces, where the 16 kHz tone was paired with the higher frequencies, the red bold lines indicate larger MMN for the higher tones than the MMN for the 16 kHz tone indicated by black bold lines (Figure 4A). In contrast, in the exposed group, where the empirical rarity of the 16 kHz tone decreased through repeated presentation, the MMN response elicited by the exposed tones paired with the lower tones became smaller than the MMN for paired lower tones (left three traces in Figure 4B) while the MMN elicited by the exposed tones paired with the higher tones remained smaller than the paired tones (right three traces in Figure 4B).

For statistical comparison, we pooled these amplitudes in four groups, e.g., (1) the lower tones (8.0, 10, and 13 kHz) and (2) 16-kHz (exposed tone for the exposed group) in the pair of 16-kHz and lower frequency tones, and (3) 16-kHz and (4) the higher tones (20, 25 and 32 kHz) in the pair of 16-kHz and higher frequency tones (Table 1, Figure 4C), and also pooled the asymmetry index in two groups, i.e., tone pairs of (1) 16-kHz and the lower tones and of (2) 16-kHz and the higher tones (Figure 4D). For the MMN amplitude, three-way ANOVA showed significant effects for the Relative height of frequency paired with 16 kHz [*F*_(1, 160)_ = 13.03; *p* = 0.00041] and for the two-way interaction Relative height × Direction of change [*F*_(1, 160)_ = 5.34; *p* = 0.022], and Direction of change × Group of rats [*F*_(1, 160)_ = 5.32; *p* = 0.022], and for the three-way interaction for Relative height × Direction of change × Group of rats [*F*_(1, 160)_ = 12.56; *p* = 0.00052]. For the asymmetry index, two-way ANOVA showed significant effects for the Relative height of frequency paired with 16 kHz [*F*_(1, 160)_ = 6.21; *p* = 0.00051] and for the two-way interaction Relative height × Group of rats [*F*_(1, 160)_ = 6.91; *p* = 0.00021]. *Post-hoc* Wilcoxon signed-rank tests revealed the frequency-dependent asymmetry of MMN in the naive group and a reversed or maintained asymmetry for the tone pairs of the exposed tone and the lower tones or the higher tones, respectively, in both the MMN amplitude (Figure 4C; naive group, *p* = 0.0025 and 0.027; exposed group, *p* = 0.0012 and 0.0031), and the asymmetry index (Figure 4D; naive group, *p* = 0.0025 and 0.017; exposed group, *p* = 0.0016 and 0.0056). *Post-hoc* Wilcoxon rank-sum tests supported the significant decrease of the MMN amplitude for the exposed tone paired with the lower tones (Figure 4C; *p* = 0.0019) and significant decrease of the asymmetry index for tone pairs of 16 kHz and the lower tones (Figure 4D; *p* = 0.000017). These results support our first hypothesis that MMN encodes the empirical rarity of sounds.

**Figure 5:**
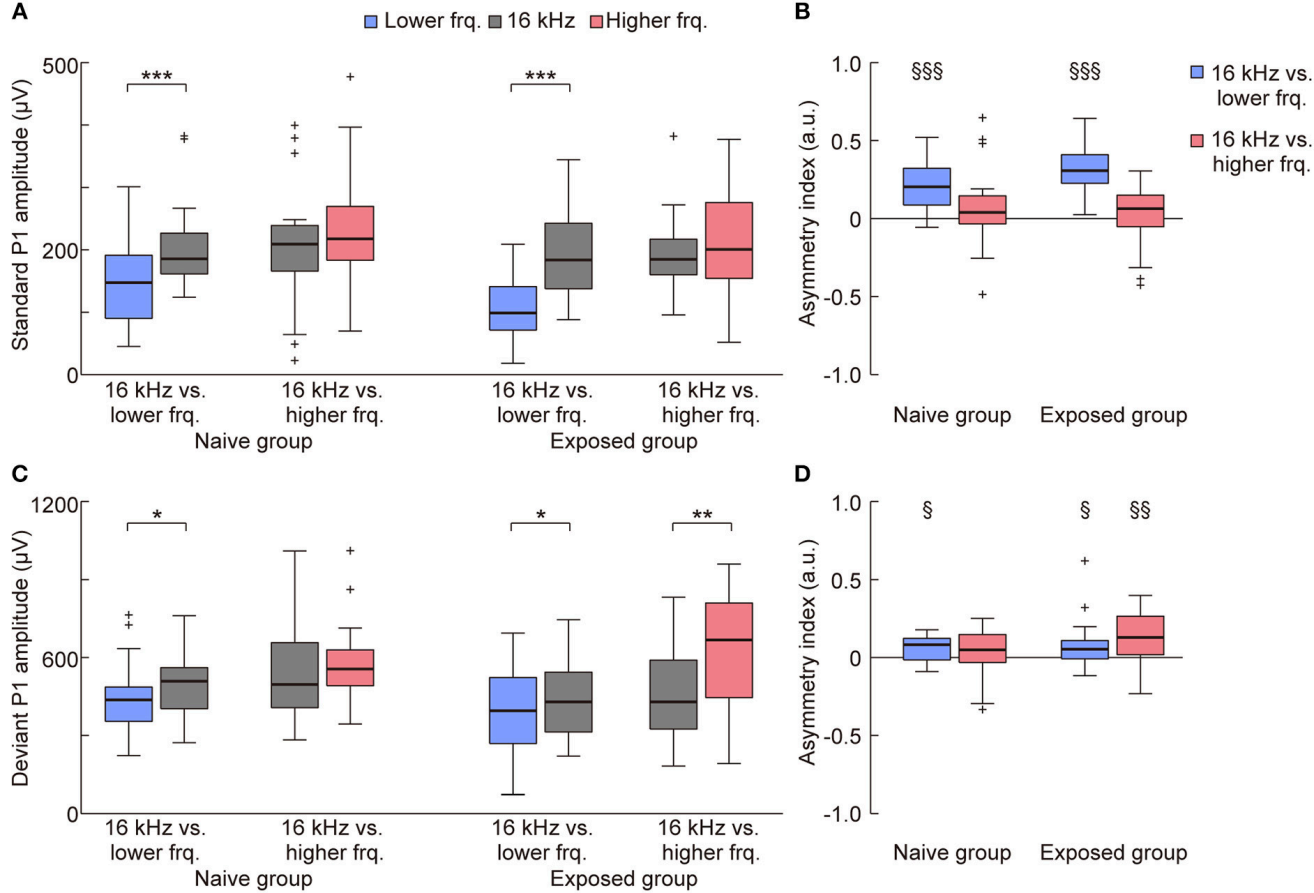
Mere exposure had no effect on the asymmetry of the P1 amplitude. The amplitude and asymmetry index of the **(A,B)** standard and **(C,D)** deviant P1 in each condition (*n* = 7 per group × 3 tone pairs) is shown. **(A)** For the standard P1 amplitude, three-way ANOVA showed significant effects for the Relative height of frequency paired with 16 kHz (exposed tone for the exposed group) [*F*_(1, 160)_ = 17.59; *p* = 0.00024], Direction of change [*F*_(1, 160)_ = 14.13; *p* = 0.000045], and Group of rats [*F*_(1, 160)_ = 7.16; *p* = 0.0082], and for the two-way interaction Relative height × Direction of change [*F*_(1, 160)_ = 5.69; *p* = 0.018]. *post-hoc* tests revealed that the amplitude responding to the lower tone was smaller than 16 kHz in the same tone pairs. **(B)** For the asymmetry index of the standard P1 amplitude, two-way ANOVA also showed significant effects for Relative height of frequency paired with 16 kHz [*F*_(1, 80)_ = 25.34; *p* = 0.0000029] and for the two-way interaction Relative height × Group of rats [*F*_(1, 80)_ = 4.48; *p* = 0.037]. *post-hoc* tests revealed the frequency-dependent asymmetry in the tone pairs of 16 kHz and lower frequencies. **(C)** For the deviant P1 amplitude, three-way ANOVA showed significant effects for Relative height of frequency paired with 16 kHz [*F*_(1, 160)_ = 5.49; *p* = 0.020], and Direction of change [*F*_(1, 160)_ = 16.45; *p* = 0.000078]. *post-hoc* tests revealed the asymmetric amplitude in three conditions. **(D)** For the asymmetry index of deviant P1, two-way ANOVA showed significant effects for Group of rats [*F*_(1, 80)_ = 3.98; *p* = 0.049]. Most importantly, *post-hoc* Wilcoxon two-sided rank-sum tests found no significant difference between groups in both the amplitude and asymmetry index (*p* > 0.05), indicating that tone exposure caused no plasticity in the P1 amplitude after exposure, while it caused rapid reversal of the frequency-dependent asymmetry of mismatch negativity. Statistical significance: ^*^p < 0.05; ^**^p < 0.01; ^***^p < 0.001 (Wilcoxon two-sided signed-rank test), ^§^*p* < 0.05; ^§§^*p* < 0.01; ^§§§^*p* < 0.001 (Wilcoxon two-sided signed-rank test vs. zero). Lower frq, lower frequency; Higher frq, higher frequency.

On the other hand, such plasticity through tone exposure was not observed in the standard and deviant P1 (Figures 4A,B, 5). The representative traces exhibit that the standard and deviant P1 amplitudes were larger in the higher tones, i.e., the black traces (16 kHz tone) in the left three traces and the red traces in the right three traces. Three-way ANOVA for the P1 amplitude did not show a tone-exposure-depending plasticity effect, i.e., the three-way interaction for Relative height × Direction of change × Group of rats. *Post-hoc* Wilcoxon signed-rank tests revealed the strong frequency-dependent asymmetry of the standard P1 amplitude in the tone pairs of 16-kHz and lower tones (Figure 5A; naive, *p* = 0.00053; exposed, *p* = 0.00011), and the weak asymmetry of deviant P1 (Figure 5C; naive, *p* = 0.027; exposed, *p* = 0.048 and 0.0019), as well as in the asymmetry index (Figure 5B; naïve, *p* = 0.00069; exposed, *p* = 0.00013; Figure 5D; naïve, *p* = 0.025; exposed, *p* = 0.040 and 0.0070). However, there was no significant change in both amplitude and the asymmetry index of standard and deviant P1, demonstrating that experience-dependent plasticity in the frequency-dependent asymmetry is limited to MMN.

### Classical-Conditioning-Induced Plasticity in MMN

**Figure 6:**
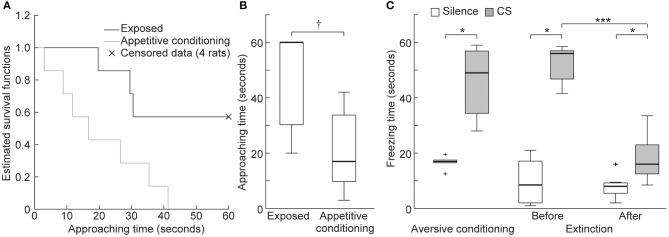
Behavioral experiments. **(A)** Kaplan-Meier survival functions to quantify approaching behavior to the food dispenser during the conditioned stimulus (CS) presentation. The four animals in the exposed group that did not exhibit poking during the 60 s presentation of the CS are indicated as censored data (× marks). The null hypothesis that there is no difference between the two groups in the probability of poking at any time point was rejected (log-rank test, *p* = 0.038). **(B)** Approaching time of the exposed and appetitive conditioning groups. Statistical significance: ^†^*p* < 0.05 (Wilcoxon rank sum test, *n* = 7 per group). **(C)** Freezing times in the aversive conditioning and extinction groups. Statistical significance: ^*^p < 0.05; ^***^p < 0.001. (Wilcoxon signed-rank test, *n* = 7 per group).

To verify appetitive conditioning, the approaching time to the food dispenser was compared between the exposed and appetitive conditioning groups. Because there were four exposed rats that did not exhibit nose poking to the dispenser during the 60 s conditioned stimulus (CS) presentation (i.e., censored data), we compared Kaplan-Meier survival functions of their approaching time (*n* = 7 per group). Consequently, there was a significant between-group difference in approaching time (Figure 6A, log-rank test, *p* = 0.038), whereby the approaching time was shorter in the appetitive conditioning group (Figure 6B, Wilcoxon rank-sum test, *p* = 0.023), indicating successful appetitive conditioning.

After aversive conditioning (Day 2), the 16 kHz CS presentation led to significantly longer freezing times than did silence in the aversive conditioning and extinction groups (Figure 6C, *n* = 7 per group, Wilcoxon sign-rank test, *p* = 0.016 for both groups). After extinction training (Day 5), freezing time during the CS was significantly shorter but was still significantly longer than that seen during silence (Wilcoxon sign-rank test, *p* = 0.016 for both comparisons). These results indicate that aversive conditioning and extinction training were successful.

**Figure 7:**
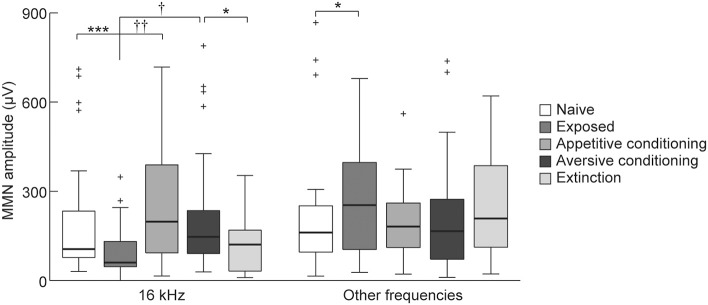
Plasticity of mismatch negativity (MMN) amplitude. MMN amplitude for each animal group (*n* = 7 per group × 6 tone pairs). For the exposed group, 16-kHz tone was exposed and for the two conditioning groups and the extinction group, 16-kHz tone was used as the conditioned stimulus (CS). Statistical significance: ^*^p < 0.05; ^***^p < 0.001 (Wilcoxon two-sided rank sum test); ^†^*p* < 0.05; ^††^*p* < 0.01 (Games-Howell test for the exposed, appetitive-conditioned, and aversive-conditioned groups).

To investigate our second hypothesis that the MMN amplitude will increase after association of a sound with salient events (i.e., appetitive or aversive classical conditioning), we compared the MMN amplitude elicited by the 16 kHz CS tone among the exposed, appetitive conditioning, aversive conditioning, and aversive-extinction groups. The appetitive and aversive conditioning groups received CS as many times as the exposed group. This repetitive presentation of CS decreased its empirical rarity and diminished the MMN responses to the CS but enhanced it at the other frequencies in the exposed group compared to the naive group (Figure 7, Wilcoxon two-sided rank sum test, *p* = 0.00069 and 0.047). While the empirical rarity of the CS equally decreased in these three groups, the rats in the appetitive conditioning and aversive conditioning groups associated the CS with salient events, i.e., food delivery and foot shock, respectively. Consequently, both the appetitive and aversive conditioning groups exhibited significantly larger MMN amplitudes than did the exposed group (Figure 7, Games-Howell test, *p* < 0.01 and *p* < 0.05), indicating that the association of sound with salient events enhances the MMN amplitude. Furthermore, extinction training after aversive conditioning significantly reduced the amplitude of MMN as compared to the aversive conditioning group (Figure 7, Wilcoxon two-sided rank sum test, *p* = 0.020). The extinction training repeatedly presented the CS to diminish its empirical saliency through both dissociation of the aversive event from the CS and decrease of the empirical rarity of the CS. Taken together with the fact that such conditioning- and extinction-induced plasticity was not observed in MMN responding to non-CS tones, these results support our second hypothesis that MMN encodes the empirical association of sound with salient events.

Conversely, there were no differences in the MMN amplitude between the appetitive and aversive conditioning groups (Figure 7). While it is beyond our current objective to reveal the representation of MMN for sound-associated emotional valence, this result suggested that a simple comparison of the MMN amplitude may not contribute to the discrimination and identification of the emotional valence of sound.

## Discussion

In this study, we investigated whether and how experience induced plasticity in tone-evoked MMN. To this end, we manipulated the empirical salience of a 16 kHz tone according to (i) sound exposure, whereby an increase in exposure causes the empirical rarity of the sound to decrease, and (ii) classical conditioning and extinction training, whereby the association and dissociation of the tone with a salient event increases or decreases the empirical importance of the sound. After sound exposure, the MMN amplitude significantly decreased and the frequency-dependent asymmetry of the MMN amplitude reversed, which indicates that MMN reflects the empirical rarity of sound. In addition, the MMN amplitude was comparably large after classical appetitive and aversive conditioning compared to mere exposure, and extinction training after aversive conditioning significantly reduced the MMN amplitude, which is consistent with our second hypothesis. To our knowledge, this is the first animal study to show that MMN reflects the empirical salience of sound in an experience-dependent manner and thus provides compelling support for the use of MMN as a biomarker of schizophrenia within the framework of the aberrant salience hypothesis.

### Empirical Salience of Sound and MMN

Consistent with our hypotheses, sound exposure decreased the MMN amplitude, and the associative learning and extinction training increased and decreased the MMN amplitude, respectively. This indicates that the MMN response reflects the empirical salience of sound irrespective of the emotional valence of sound. Our results showed that MMN and the anterior insula share a similar activation pattern, which is responsive to stimulus salience, i.e., dependent on probability and experience but irrespective of its emotional valence (Downar et al., 2000, 2002; Craig, 2002; Sridharan et al., 2008; Gogolla, 2017). However, we cannot conclude that the MMN response is indicative of salience-detecting neural activity preceding the salience network because a causal relationship between the auditory cortex and anterior insula during deviance processing has not yet been elucidated (Sridharan et al., 2008). On the other hand, taken together with the previous observation that MMN spread toward the belt area, dense projections from the belt area of the auditory cortex to the insula support the possibility that the MMN response reflects hierarchical salience processing from the sensory cortex to the salience network (Munoz-Lopez et al., 2010; Palaniyappan et al., 2013).

### Frequency-Dependent Asymmetries of MMN and P1 Responses

The MMN amplitudes of the naive group exhibited a frequency-dependent asymmetry whereby incremental frequency changes elicited larger MMN responses than decremental changes with the same tone pair in the oddball paradigm. A similar asymmetry has been reported in a human study (Peter et al., 2010), yet its behavioral implications remain unclear. We hypothesized that this MMN asymmetry represents the empirical rarity of sound that is primarily acquired from the natural acoustic environment, in which low-frequency noises are dominant. As expected, exposure to a 16 kHz tone resulted in a decreased MMN amplitude and reversed the asymmetry of the MMN elicited by the tone pairs of 16 kHz tone and tones lower than 16 kHz. Taken together, our findings indicate that the MMN amplitude reflects the empirical rarity of sound, which can be altered by a few hours of exposure and maintained for several days.

While the amplitude and frequency-dependent asymmetry of the MMN response exhibited plasticity, those of the standard and deviant P1 did not exhibit drastic change after the sound exposure. There was no significant difference in amplitude between the naive and exposed groups, and their asymmetries were weak and not reversed after sound exposure. We reasoned that the frequency-dependent asymmetry of the P1 amplitude may reflect the asymmetric shape of tuning curves in the mammalian auditory cortex, thus remaining unchanged after harmless sound exposure using a 60 dB tone. One previous study reported that the frequency-responsive area is often larger for low frequencies than for high frequencies; neurons were thus more responsive to the lower frequencies (Sutter, 2000). This asymmetry of responsive areas signifies that there is stronger adaptation to lower tones (Brosch and Schreiner, 1997), which may explain the smaller standard and deviant P1 to the decremental frequency change compared to the incremental change. In addition, the reported difference in the spatial distribution of the P1 and MMN response [the P1 tonotopically elicited from the core area, while the MMN spreading toward the belt area without tonotopic structure; (Shiramatsu et al., 2013)] may indicate that the frequency-dependent asymmetry of MMN is mediated by top-down mechanisms for deviance and salience detection rather than by the asymmetry in frequency tuning.

The equivalent P1 amplitude between the naive and exposed groups also provides preliminary evidence for the difference between SSA and MMN. The present study showed the robustness of the P1 amplitude against sound exposure, indicating that the SSA defined by P1 is independent from the empirical rarity of sound. Under the oddball paradigm, SSA works as a deviance detector or surprise indicator as well as MMN by adapting the response to repetitive standard and highlighting the response to deviants (Ulanovsky et al., 2003). However, accumulating evidence has indicated the difference between SSA and MMN; SSA mainly depends on bottom-up neural substrates, while MMN is mediated by both bottom-up and top-down neural processing (Farley et al., 2010; Fishman and Steinschneider, 2012; Shiramatsu et al., 2013). In our recording, P1 mainly reflects thalamocortical input responding to sound onset, which depends on the responsiveness of neurons in the bottom-up auditory pathway. Mere exposure to a moderate (not too intense) sound could not change this neural responsiveness, which is one of the reasons why the P1 amplitude and SSA did not represent the empirical rarity of sound. Besides, the amplitude and frequency-dependent asymmetry of MMN drastically changed according to the empirical rarity of sound, indicating a stronger top-down effect on MMN than on P1 and supporting the difference between MMN and SSA.

### Emotional Valence and MMN

While this study showed that both appetitive and aversive classical conditioning enhanced the MMN response, there was no difference in MMN amplitude between these two types of conditioning. This indicates that MMN reflects the primitive recognition of emotional salience of deviant sounds, but without distinguishing between different emotional valences. The early latency of the MMN response (100–300 ms in humans) supports this notion, in that the emotional valence of sound is reportedly first represented at approximately 1,500–3,000 ms post-stimulus onset, as reflected by differences in the later EEG components (Erhan et al., 1998). Conversely, these findings do not exclude the possibility that MMN reflects early processing of the emotional valence of sound because it is not clear whether the arousal level of the appetitive and aversive US was equal (Martin-Soelch et al., 2007). An additional study that would quantitatively calibrate (Ilango et al., 2010) the influence of these reinforcers may elucidate this.

### Eligibility of MMN as a Biomarker in the Aberrant Salience Hypothesis of Schizophrenia

MMN has been widely considered a major candidate biomarker in the glutamate hypothesis of schizophrenia, which regards the dysfunction of NMDA receptors as a major cause of schizophrenia. Besides, the present results suggested that MMN represents empirical salience and provided the first empirical evidence that MMN is eligible as a biomarker in the aberrant salience hypothesis of schizophrenia emerged in the dopamine hypothesis of schizophrenia. The aberrant salience hypothesis attempts to link the disturbances of attention and experience-based predictions in schizophrenia (Hemsley, 1998, 2005a,b; Nelson et al., 2014) with gray matter abnormalities in the anterior insula (Ellison-Wright et al., 2008; Glahn et al., 2008) and functional connectivity with the salience network (White et al., 2010; Moran et al., 2013; Orliac et al., 2013; Palaniyappan et al., 2013; Manoliu et al., 2014). This nucleus and network contribute to attention switching and are activated in response to experience-based deviant stimuli in the oddball sequence (Downar et al., 2000, 2002; Sridharan et al., 2008; Palaniyappan et al., 2013). The present study suggests a link between MMN and empirical salience and raises the possibility that MMN represents aberrant salience processing in schizophrenia patients, which will establish the eligibility of MMN as a biomarker of the aberrant salience hypothesis of schizophrenia.

We expect further studies to test this possibility. For example, the correlation between MMN amplitudes and the mimicked symptoms mediated by the hyperactivation of dopaminergic receptors should be examined in animal models, and changes in MMN amplitudes after medication should be investigated irrespective of the neurotransmitters targeted. Such future studies will link the impaired MMN as described in the glutamate hypothesis (Shelley et al., 1991; Baldeweg et al., 2004; Brockhaus-Dumke et al., 2005; Light and Braff, 2005) and impaired salience processing as discussed in the dopamine hypothesis of schizophrenia (Kapur, 2003; Nelson et al., 2014; Uddin, 2015), establishing MMN as a strong biomarker of schizophrenia.

## Conclusion

To test the potential of MMN as a clinical biomarker of schizophrenia, the present study investigated whether MMN encodes the empirical salience of sound, which depends on both the (i) empirical rarity of sound and (ii) association of sound with salient events through classical conditioning. Consequently, the frequency-dependent asymmetry of the MMN amplitude reversed after sound exposure, supporting our first hypothesis. In addition, our second hypothesis was verified by the enhanced and diminished MMN amplitude after classical conditioning and fear extinction, respectively. These findings suggest that MMN encodes the experience-based salience of sounds and will enable further behavioral studies to investigate the link between impaired MMN as described in the glutamate hypothesis and impaired salience processing as discussed in the dopamine hypothesis of schizophrenia and to establish MMN as a strong biomarker of schizophrenia.

## Author Contributions

TS and HT designed the research, analyzed the data, interpreted the results, and approved the final version of the manuscript. TS performed the experiments, prepared the figures, and drafted the manuscript. HT revised the manuscript.

### Conflict of Interest Statement

The authors declare that the research was conducted in the absence of any commercial or financial relationships that could be construed as a potential conflict of interest.
